# Experiences of Women Who Have Had Carrier Testing for Duchenne Muscular Dystrophy and Becker Muscular Dystrophy During Adolescence

**DOI:** 10.1007/s10897-018-0266-0

**Published:** 2018-07-04

**Authors:** Harry G. Fraser, Rebecca Z. Redmond, Diana F. Scotcher

**Affiliations:** 1 Division of Evolution and Genomic Sciences, School of Biological Sciences, University of Manchester, Manchester, UK; 2 Manchester Centre for Genomic Medicine, St Mary’s Hospital, Central Manchester University Hospitals NHS Foundation Trust Manchester Academic Health Sciences Centre, 6th Floor, Oxford Road, Manchester, M13 9WL UK

**Keywords:** Duchenne muscular dystrophy, Becker muscular dystrophy, Carrier testing, Adolescence, Genetic counseling, X-linked, Psychosocial, Qualitative

## Abstract

Duchenne muscular dystrophy (DMD) and Becker muscular dystrophy (BMD) are X-linked recessive degenerative muscular conditions. Carrier testing is available to at-risk females. Though carrier testing is often offered to adolescent females, it raises ethical issues related to autonomy. This study aimed to address the impact of DMD/BMD carrier testing during adolescence, to elucidate what motivates adolescents to seek testing, and to assess the carrier testing experience. Retrospective semi-structured telephone interviews were conducted with 12 women out of 28 initially contacted. Data were coded using thematic analysis. For most (8/12) participants, discovering their carrier status during adolescence appeared to have helped alleviate uncertainty. The majority (9/12) of participants felt that they had made an autonomous decision and most (10/12) seemed to have adjusted well to their test result. Reproductive factors were framed as having been a key motivator prior to testing. However, following testing, participants’ views on prenatal diagnosis seemed more closely linked to their lived experience than to their test result. Just over half (7/12) the participants reported having not had the opportunity for genetic counseling prior to testing and after receiving their result, an issue that warrants further consideration.

**Electronic supplementary material:**

The online version of this article (10.1007/s10897-018-0266-0) contains supplementary material, which is available to authorized users.

## Introduction

### Overview of DMD/BMD

Duchenne muscular dystrophy (DMD) and Becker muscular dystrophy (BMD) are X-linked recessive genetic conditions that are characterized by progressive muscle wastage, resulting in a gradual loss of ambulation, cardiac dysfunction (Emery 2002), and respiratory weakening that eventually leads to respiratory failure (Bushby et al. 2010). Both are caused by pathogenic variants in the *DMD* gene, which codes for the protein dystrophin. DMD affects approximately 1 in 3500 males (Manzur and Muntoni 2009) and has an earlier age of onset and a more severe prognosis than BMD, which affects approximately 1 in 18,000 males. Death typically occurs during the third decade in males with DMD and the fifth decade in males with BMD (Hermans et al. 2010; Hoogerwaard et al. 1997). Although males with BMD often go on to have children themselves, this is less common for those affected with DMD.

### X-Linked Carrier Testing in Adolescence

Most females with a pathogenic *DMD* gene variant present as asymptomatic carriers due to the presence of a second normally functioning allele. However, a proportion of female carriers will have some associated cardiomyopathy, particularly carriers of DMD (Florian et al. 2016). Each male child of a carrier female has a 50% chance of being clinically affected with either DMD or BMD, depending on the specific gene variant. Carrier testing can indicate if a woman is at risk of having affected sons, and if any additional cardiac screening is advisable. In the UK, genetic counselors are trained to support people through carrier testing. This research focuses on carrier testing during adolescence. The World Health Organization (WHO) has defined adolescence as the period of development preceding adulthood, between 10 and 19 years of age (WHO 2015).

Several potential negative implications of carrier testing during adolescence have been highlighted in the literature. These include loss of autonomy, stigmatization, and anxiety following an unfavorable test result (Elger 2010). In two studies, girls theorized that being found to be a carrier of a condition could lead to fear of rejection by a future partner (Borry et al. 2005; James et al. 2003). It has also been suggested that a result that indicates that a daughter is a carrier of a genetic condition may distort the family’s perception of her (Borry et al. 2006b).

However, there is little evidence of a harmful long-term effect of carrier testing during adolescence (Lerman et al. 2002), and any test-related anxiety tends to dissipate within 6 months (Lewis et al. 2011). A study that assessed the experiences of DMD carrier testing in adolescent girls found that the majority (42/46) retained a positive or fairly positive attitude towards the process after testing was complete, and that most (39/46) would recommend testing (Järvinen et al. 1999). In the context of fragile X syndrome, the majority (51/53) of girls tested in one study stated that they would still want to know their carrier status if they were given the choice again (Wehbe et al. 2009).

After reviewing the literature, Wade et al. (2010) concluded that there was little evidence that carrier testing had a clinically significant impact on the emotional state or self-perception of adolescents. Female fragile X carriers in one study may have actually demonstrated better coping mechanisms than untested girls who were aware that they were at risk (Wade et al. 2010). Both carrier and non-carrier results can enable an adolescent to obtain a sense of control, potentially contributing to their personal growth and maturity (Järvinen et al. 2000; McConkie-Rosell et al. 2012). Furthermore, an unfavorable test result can reduce uncertainty, enabling an adolescent girl to adjust to the knowledge of her carrier status (Elger 2010; Fanos 1997). Vears et al. (2017) have argued that there is currently no evidence that childhood carrier testing causes any harm, and therefore there is no ethically justifiable reason to refuse testing.

### Motivation for Carrier Testing

The process of information gathering has been highlighted as being of primary importance for adults, both prior to and after the testing process (Lewis et al. 2012). Even an unfavorable result can be reframed positively, providing the information necessary to make fully informed reproductive decisions (Lewis et al. 2012). This gives individuals the opportunity to take control, ensuring autonomy in the choices they make (Zaccaro and Freda 2014). From the desire for more information, responsibility, and autonomy, the concept of “reproductive empowerment” has emerged (Lewis et al. 2012).

The prominent role for autonomy and empowerment in the adult literature raises concerns over the appropriateness of performing carrier testing during adolescence. It is questionable whether adolescents are autonomous or mature enough to fully understand the implications of carrier testing, or to make the decision to undergo testing independently. The potential for jeopardizing an individual’s future autonomy is deemed to be the main issue at stake when considering the ethics of adolescent carrier testing (Borry et al. 2006a; Vears et al. 2017). While adult self-concept is perceived as being stable, the adolescent self-concept is still in a stage of development, affected by experiences and challenges (McConkie-Rosell et al. 2008). It is thus conceivable that carrier testing could negatively impact this process.

There are some examples in the literature where adolescents have been able to verbalize motives for seeking carrier testing at a younger age. Future reproductive decision-making and being able to be open in romantic relationships have been offered as factors motivating adolescent girls to seek carrier testing for X-linked and autosomal recessive conditions (James et al. 2003). A Belgian study indicated that just over half (93/166) of adolescent students who were at no known increased genetic risk would want risk information before a pregnancy (Decruyenaere et al. 1995). The students felt that testing would enable informed reproductive decision-making and provide the opportunity to become emotionally prepared for the birth of a child with a genetic condition. This indicates that they were cognitively able to make informed decisions about obtaining carrier information (albeit theoretically). Wehbe et al. (2009) reported that adolescent girls put more emphasis on the importance of properly understanding and personalizing carrier information, rather than focusing on a potential negative emotional response. Parents have reported sharing the view that their daughters’ knowledge of carrier status could help aid future reproductive decision-making (Hayes et al. 2016; Vears et al. 2016).

### Current Practice and Guidelines

In one large-scale European study, clinical geneticists agreed that testing adolescents for genetic carrier status at 15 years of age would violate their future autonomy and confidentiality (Borry et al. 2007), and that there was rarely significant justification for offering carrier testing to a 15-year-old. However, the clinicians also stated that cognitive, emotional, and sexual maturity was of greater importance than age alone, and that they assessed each case individually (Borry et al. 2008). This correlates with an American study (Multhaupt-Buell et al. 2007) which found that many requests for carrier testing in adolescents are being granted and that most (205/244) clinicians who had received test requests had offered adolescent carrier testing at least once. Factors influencing clinicians’ decisions included whether the adolescent was sexually active and whether they were perceived as being competent.

Guidelines generally recommend that requests for carrier testing should be deferred until adulthood (Borry et al. 2006a). For example, the American Society of Human Genetics (1995) has advocated the delay of carrier testing until adulthood, as the benefits of testing do not supposedly become apparent until then. However, more recent guidelines demonstrate more flexibility and recommend assessment on a case-by-case basis (British Society of Genetic Medicine 2010).

### Summary

It has been acknowledged that the majority of research assessing carrier testing has focused on the appropriate age for testing, neglecting the actual decision-making process (Lehmann et al. 2011). The research that has addressed motivational factors has generally focused on parental motives. While investigations that have directly addressed adolescent motivational factors have provided promising insights (Wehbe et al. 2009), there is a paucity of such data, little of which addresses carrier testing for DMD/BMD. Hayes et al. (2016) have illustrated the need for research assessing the reproductive choices of DMD carriers who had genetic testing during adolescence. As research into motivational factors and the impact of carrier testing in adolescence is lacking, this is an area that would benefit from further exploration.

The intention of this research study was to build on the limited existing literature and address the lack of understanding around the process of adolescent motivation for carrier testing in the context of DMD/BMD. The aim was to focus specifically on the motivational factors driving carrier test requests and the subsequent impact of test results by conducting in-depth interviews with women who received carrier testing for DMD/BMD during adolescence. The study also aimed to address patient experiences of the counseling process, given the potentially challenging and emotional nature of adolescent carrier test requests.

## Methods

### Research Questions

What motivates young women to request carrier testing during adolescence? What is the impact on a young woman of having a carrier test? What do women think about the support and counseling they received at the time of testing?

### Study Design

A qualitative semi-structured interview design was utilized for the collection of data. This was deemed appropriate due to both the highly exploratory nature of the investigation and the desire to extract rich detailed information about a complex process. The investigation was retrospective in nature, a necessity for the reflection on the long-term impact of testing. Participants were interviewed by telephone.

### Participants

Appropriate prospective participants who met the following criteria were identified through the Manchester Genetic Family Register Service database by the authors. Inclusion criteria were as follows: the participant was female, aged over 18, had carrier testing for DMD/BMD during adolescence, and a minimum of 1 year prior to the invitation being sent. Individuals with both carrier and non-carrier test results were approached. Exclusion criteria were as follows: the prospective participant did not speak English, was known to have learning difficulties, was known to be pregnant, or had already had an affected child prior to having carrier testing. Prospective participants who were known to the department to have mental health problems such as anxiety, depression, bipolar disorder, or schizophrenia were excluded. This information was ascertained through departmental clinical notes. The rationale for this was that the interviewers had limited experience in counseling individuals with mental health problems, in addition to the sensitive nature of the research. Prospective participants with a sibling who had already been invited to be interviewed were also excluded. Given the small nature of the study, it was believed that the possibility of similar experiences within sibships would skew the data.

### Procedures

Ethical approval was sought via the NHS Research Ethics Committee (REC) as part of a wider investigation entitled “Patient’s experiences of living with a genetic condition and utilizing clinical genetic services” (REC Reference: 15/NW/0013).

Telephone interviews took place in May and June 2015. The interviews were recorded, transcribed, and anonymized after completion. Interviews were carried out by two MSc Genetic Counseling students with training in thematic analysis. The interviewers were equipped with an interview guide (Appendix 1), which was designed to guide discussion rather than be prescriptive. Participants were given the opportunity to request a referral back to their genetic counselor or doctor in Genomic Medicine if they felt they would benefit from additional support following their interview.

### Data Analysis

Interviews with participants with a family history of DMD were conducted and coded by one interviewer, and interviews with participants with a family history of BMD were conducted and coded by the second. The two interviewers initially familiarized themselves with the data. The transcribed interviews were examined using thematic analysis (Aronson 1995). Thematic analysis has been advocated as a useful and flexible approach to analyzing qualitative research (Braun and Clarke 2006). The interviewers worked closely together, discussing proposed themes and assessing which most accurately represented the raw data. Data were swapped and annotated transcribed material was compared, in order to ensure that both interviewers could identify all proposed themes. At this stage, any disagreements were discussed and resolved.

## Results

The recruitment process identified a total of 28 potential participants: 14 who had carrier testing for DMD (3 carriers, 11 non-carriers) and 14 who had carrier testing for BMD (9 carriers, 5 non-carriers). Of the 28 women contacted, 12 agreed to take part in the study. The average age of participants at the time of interview was 23.6 years (range 18–34). The average age at which participants had received carrier testing was 15.4 years (range 14–17), and the reported average age at which participants had been made aware of their carrier risk was 11.4 years (range 6–14). At the time of interview, 3 out of 12 participants had unaffected children, while none had children affected with DMD/BMD.

Five main themes were drawn from the data. These themes were recurrent across both the DMD/BMD groups. Table 1 summarizes the details of the 12 participants interviewed.

**Table 1:** Summary of participant details

Participant	Condition	Age at testing	Age at interview	Carrier status	Closest affected relative (alive/deceased)	Who initiated testing discussion?	Aware of risk for how long?	Affected children	Unaffected children	Impact on reproductive decision-making?
Participant 1	DMD	17	23	Non-carrier	Brother (alive)	Mother	3 years	No	No	No
Participant 2	DMD	15	19	Non-carrier	Two brothers (both alive)	Doctor	3 years	No	No	No
Participant 3	DMD	14	18	Carrier	Brother (alive)	Mother	2–4 years	No	No	Uncertain
Participant 4	DMD	17	34	Non-carrier	Brother (deceased)	Mother	Not reported	No	4	No
Participant 5	DMD	16	21	Non-carrier	Brother (alive)	Parents	3 years	No	No	Yes, feels more positive about reproductive future
Participant 6	BMD	15	32	Carrier	Uncles (deceased)	Mother	< 1 year	No	No	Yes, delayed having children, would terminate affected pregnancy
Participant 7	BMD	16	18	Carrier	Father (alive)	Father	3 years	No	No	Uncertain
Participant 8	BMD	14	18	Carrier	Brother (alive)	Mother	2 years	No	No	No
Participant 9	BMD	14	23	Carrier	Brother	Mother	6 years	No	No	Uncertain
Participant 10	BMD	17	28	Carrier	Half-brother (participant adopted)	Parents	7 years	No	1	Fetal sexing—female
Participant 11	BMD	16	26	Non-carrier	Brother (alive)	Genetics (letter)	10 years	No	No	Uncertain
Participant 12	BMD	14	23	Carrier	Cousin (alive)	Mother	1–2 years	No	1	Fetal sexing—female

### Motivation to Discover Carrier Status

Most (8/12) participants described at least one personal incentive for having carrier testing. Some stated that not knowing was more difficult than having either a confirmed carrier or non-carrier result.

“I think it made me more certain I wanted to know, because the more and more we talked about either/or ... I became really anxious to know, so I could, I could formulate some kind of thought because at that moment I was just split in two, I could be this I could be that.” [participant 5, DMD, non-carrier]

When obtaining certainty was not a central issue (where a participant was an obligate carrier of BMD due to her father being affected), there was instead a symbolic significance attached to having carrier testing.

“I just wanted to know for certain like. I already knew I’d be having it. I think I just wanted… my limelight! I can’t find the words. I just wanted to know for certain, find out if it’s for sure.” [participant 7, BMD, carrier]

With the increased likelihood of sexual contact, it was deemed sensible to have testing “just in case,” a view shared by both mothers and daughters. For participant 1, this was prompted by a first relationship.

“Just in case anything ever does happen, erm in case you do become pregnant, we do need to know about all the possibilities and do I actually carry the gene ... it’s a big thing to have an impact on your life, so I would need to know if my child could have that gene.” [participant 1, DMD, non-carrier]

Participant 5 discussed thoughts about the future and developing a more adult perception of the world.

“It was such an adult thing for a 14-year-old to be considering, but I think when you’re at that age you’re just starting to see the world like as an adult, you can start to see yourself having kids.” [participant 5, DMD, non-carrier]

Throughout the carrier testing process, parents, particularly mothers, were found to have an influential role in the course of proceedings.

“It was my mum that gave me the opportunity to do it and then she said ‘do you want to know?’ and I said ‘well now you’ve asked me yeah! I can’t not know now!’” [participant 6, BMD, carrier]

Participant 1 presented her mother’s opinion as holding greater weight than the views of healthcare professionals.

“I think, at 17 I would have only valued my mum’s opinion … I was like, I hate everyone, I hate everything that has happened to us, so I don’t think the medical profession you know, I’m not saying this in a bad way but they don’t live it, they know about it but they don’t actually live with it.” [participant 1, DMD, non-carrier]

### Positive Attitude Towards Testing

Most (10/12) participants expressed a positive attitude towards having had carrier testing during adolescence, and most felt that having testing at this time had felt an appropriate, autonomous decision.

“My mum would’ve never forced me into it ... I do feel I was the controller of the decision, it was my decision, it was me who said I wanted the test.” [participant 1, DMD, non-carrier]

On the whole, participants felt that they had been provided with enough information to give fully informed consent to undergo testing.

“I understood completely ... everything that was going to happen to me.” [participant 5, DMD, non-carrier]

A non-carrier test result enabled participant 2 to find who she was on a personal level, having grown up living with two brothers affected with DMD.

“Growing up it was quite difficult because all the attention was on my brothers ... I never really found who I was and that, and I never really did till I [had testing] ... it was a lot easier to erm to try and find myself personally really knowing that … It pushed me to helping those who weren’t as lucky as me really.” [participant 2, DMD, non-carrier]

Participant 9 believed that growing up with the knowledge about her carrier status from a young age has been helpful.

“I think then it would be easier for me to accept than it would be now. Now I’ve kind of had, do you know what I mean? Kind of a chance to accept it. Knowing that it’s a possibility that I could have children with muscular dystrophy.” [participant 9, BMD, carrier]

One participant reported a belief that being at a younger age, and thus lacking experience and understanding, acted as a protective buffer against potential bad news.

“Yeah, I don’t think it was a bad thing, I don’t think I took it badly, I feel like if anything I didn’t understand like as much as I do now so I’d probably take it worse now than when I was younger.” [participant 3, DMD, carrier]

There were some exceptions to the consensus that testing during adolescence had been the right thing to do.

“I think [testing is better] as an adult. As much as you think you really know about things when you’re a teenager. When you look back maybe you weren’t quite as sure as you thought you were.” [participant 11, BMD, non-carrier]

### Limited Significance of Test Results

For half (6/12) the participants, carrier test results appeared to have a modest impact on future reproductive plans. Participant 2 described a non-carrier test result as initially taking “a massive weight off my shoulders”, but as time passed her result felt less important.

“Thinking more I’ve like realized that it doesn’t really matter and the situation’s what you make it ... and even if the results were different now, it wouldn’t really affect me I don’t think.” [participant 2, DMD, non-carrier]

Participant 3, the only DMD carrier, cited other practical factors that may influence future reproductive decisions.

“I guess obviously you’d have to be sorted for money and a house and everything.” [participant 3, DMD, carrier]

For carriers of BMD, there was a focus on the perceived severity of the condition.

“Personally I don’t believe in getting rid of them … Don’t get me wrong. If it was like serious. If the baby was born seriously brain, you know, serious problems? [sic]” [participant 8, BMD, carrier]

Another participant spoke about her personal experience of BMD.

“I think it wasn’t worrying because I know [affected cousin] has always been fine and although he’s got a condition and, and what have you, it’s never held him back or anything.” [participant 12, BMD, carrier]

However, for some (4/12) participants, it was apparent that carrier test results had been reproductively significant.

“I’ve always wanted a big family even when I was little and I remember wanting a big family but this couple of year period of my life it seemed that I might not be able to have that ... [The future] just became a lot more exciting, something to look forward to.” [participant 5, DMD, non-carrier]

Participant 6 felt she would want to intervene in a future pregnancy.

“I will abort it if it’s [an affected] boy … I just think that when you’ve seen your family and you know, my uncle passed away, my cousin. I don’t think I could ever go through that as a mum. I wouldn’t ever want that for my child.” [participant 6, BMD, carrier]

Participant 10 reflected on how her carrier status had an impact on her reproductive decision-making.

“I’d been wanting to get pregnant for quite a while. That was always at the back of my mind, you know, what if I ended up having a boy and you know he has muscular dystrophy … So I think in a way it probably did stop me from having children sooner.” [participant 10, BMD, carrier]

### Need for In-Depth Psychosocial Discussion

Though participants on the whole reported that they had received enough information to enable them to make an informed decision to opt for carrier testing, just over half (7/12) felt there was a lack of psychosocial exploration of the possible implications of carrier testing. Participant 11 believed that she would have benefited from a second appointment.

“I think, more than one session. I went for the counseling and then at the end of the counseling session they took the blood test … I think it would have been better to go and talk for one about it and then go away and think about it. It was more first appointment yes or no.” [participant 11, BMD, non-carrier]

Participant 4 did not recall having had any genetic counseling prior to testing.

“I didn’t have any counseling … I didn’t [know I could have seen a genetic counselor], they just said it was a blood test, and I just said, ‘okie doke’.” [participant 4, DMD, non-carrier]

Some participants spoke about the difficulty involved when disclosing a carrier result to a partner.

“My biggest worry is if I get someone, like when do I tell them? … I do worry if anyone’s going to be accepting that there is a chance they’ll have a son with a disability. … That’s what I worry the most about: telling them and then they won’t want to have children with me.” [participant 9, BMD, carrier]

Some (4/12) participants reported having had more in-depth counseling and expressed greater satisfaction with the process.

“[The counselor] didn’t explain to me in a load of scientific jargon, she just talked to me calmly about how I feel about having a brother with a condition like this and how I’d feel if I was to be a carrier of it ... I didn’t even know I knew so much I think, but because she approached it in such a sensitive way, I felt very calmed, like I just opened the floodgates really, and I think that’s like the only time I’ve calmly spoken about it.” [participant 5, DMD, non-carrier]

She explicitly separates this process from the scientific aspects of the discussion:

“I think it was good to have the science basis, but ... to have the more emotive, the more feeling kind of, process with the counselor really helped.” [participant 5, DMD, non-carrier]

### The Importance of Follow-up

Half (6/12) the participants commented on the value of long-term follow-up. Some who had non-carrier results felt that they were not given the opportunity for follow-up.

“I don’t know if I should have had any follow-up. It was just put in a letter and that’s it … Some people might not deal well with finding out that they aren’t a carrier, maybe [a phone call] a bit later on just to, you know, check that everything’s OK.” [participant 11, BMD, non-carrier]

Participant 1 reported feeling as if she was “left to it” after a non-carrier result. She acknowledged that it would not have been until she was older that she would have taken up an invitation for follow-up.

“I would have benefited from some more, at a later age, like when I got to 20 … but I think at 17/18 I would have disregarded it, disregarded any offer of counseling then but at 20, 21 I would have snatched their hand off for it,” [participant 1, DMD, non-carrier]

Participant 6 recalls being informed of her result, but felt that she did not fully process it at the time.

“It’s processing the information that you can’t deal with at that age. You can be told the result and sit there and agree and nod your head, but if it actually goes into your head! … I probably do wish that I would have years ago maybe talked about it to someone like yourself … Because then I might have, my life might have planned out a bit different,” [participant 6, BMD, carrier]

## Discussion

This study explored the experience of DMD/BMD carrier testing during adolescence, and to our knowledge is the first study to have directly addressed the motivational factors driving adolescent requests for carrier testing for these conditions. Though our research indicates that the women interviewed were generally satisfied with having had carrier testing during adolescence, there were some issues with the carrier testing process identified, particularly in the post-test follow-up period.

### Motivation to Discover Carrier Status

In this study, participants offered several reasons why testing could be beneficial during adolescence. One key motivational factor was the alleviation of uncertainty. This is supported by previous research (Mand et al. 2013) where participants described the period of not knowing their carrier status as difficult and anxiety provoking.

Another motivational factor related to new relationships or a concern regarding an unplanned pregnancy, and there was also evidence of a need to obtain carrier knowledge in order for participants to have control over future reproductive options. This is in parallel to literature assessing parental motives for adolescent carrier testing, where parents held a perceived responsibility to make their daughters aware of any increased risk of a genetic condition before reproduction (James et al. 2003; McConkie-Rosell et al. 1999).

Parents, mothers in particular, had a key role in initiating discussions with their daughter about carrier testing. This could indicate a sense of perceived maternal responsibility due to the origin of the familial *DMD* gene variant, reflecting the maternal guilt that has been reported in the literature on X-linked conditions (Lewis et al. 2011). Alternatively, it may reflect the more general strength of the mother-daughter bond, and the perceived maternal role of emotional caregiver. Participants who felt that their parents had been centrally involved in their decision did not express any resentment or dissatisfaction towards their parents for this. Instead, they appreciated this input.

### Positive Attitude Towards Testing

The majority of participants felt that adolescence had been the appropriate time for carrier testing. Although participant 3 (DMD, carrier) felt that having testing during adolescence made her carrier result easier to manage, there is some indication that her age may have limited her ability to make a fully informed decision at the time of testing. Her comments suggest that she now has a better understanding, but it is unclear whether this is the result of information-seeking post-carrier test, or if it is due to increasing maturity levels over time and hence a greater understanding of the implications of testing. This response could be interpreted as an indication that bad news may be easier to take as an adolescent than as an adult, partly due to participants not understanding the full implications of their result during adolescence. This is in contrast to findings by James et al. (2003), where teenage girls suggested that carrier testing for X-linked conditions was more appropriate in adulthood, because they would not be able to cope with a result during adolescence.

Participants generally reported having a strong sense of autonomy over the decision to have carrier testing. This differs from previous research that found that many girls feel they are denied a say in the decision-making process for DMD carrier testing (Järvinen et al. 1999). However, the extent of this autonomy appears to be somewhat at odds with the reported parental influence. It may have been that parents and daughters shared the same desire for testing, or that daughters were at ease with their parents taking a central role.

There was no evidence of carrier testing being detrimental to the development of the adolescent self-concept. This is comparable to findings from Wade et al. (2010) and McConkie-Rosell et al. (2008), where fragile X carrier testing in adolescence had little negative impact on self-perception.

### Limited Significance of Test Results

Reported lived experience of DMD/BMD appeared to have impacted participants, and there was evidence that personal experience of the condition and carrier status acted as independent predictors of future reproductive plans.

While the gathering of reproductive information was frequently presented as a motivational factor for undergoing carrier testing, more emphasis was subsequently given to gaining knowledge about the self and relieving uncertainty. This creates what we have labeled a “pregnancy paradox,” in which test results have an apparently limited impact on future reproductive decisions, despite this initially being framed as a key motivator for testing. It may be that when initially attempting to deconstruct the testing process, making reference to a clear practical reason for testing helped make the decision easier. However, other studies have found a strong impact of DMD carrier test results on reproductive plans (Eggers et al. 1999; Emery et al. 1972). Due to the age range in the present study, it is conceivable that the participants may have different opinions regarding their reproductive plans in the future. It is also possible that because the majority of participants with a family history of DMD were non-carriers, it may have been difficult for them to conceptualize the potential significance of an unfavorable test result. This is thus a concept that warrants further investigation.

Our research supports a body of literature that suggests that personal experience of a genetic condition is potentially as relevant as an individual’s carrier status (Eggers et al. 1999; Raspberry and Skinner 2011; Nabukera et al. 2013). Carrier status may be just one of many factors influencing reproductive decision-making.

### Need for In-Depth Psychosocial Discussion

Potential explanations for the lack of counseling input described by some participants include that more in-depth counseling may have been available but participants chose not to or were unable to engage with this, and that while as adults these participants retrospectively value the input of a counselor, this may not have been the case during adolescence. This finding from our research echoes concerns highlighted by Järvinen et al. (2000), who suggested that carrier testing at a young age may deter people from requesting genetic counseling.

Participants spoke about the challenges of disclosing results to partners. This has been reported previously by women who are carriers of X-linked conditions (Kay and Kingston 2002) and suggests that some women may fear that disclosing X-linked carrier status could discourage a partner from committing to a relationship. This issue highlights the potential value of early discussions and input from a genetic counselor.

For participant 5 (DMD, non-carrier), who reported having received in-depth counseling, the benefits of this were clear. The sensitive approach was personalized, which gave her the opportunity to compose herself and provided a platform on which she could open up and express her concerns. Though scientific information is necessary, on its own, it is inadequate without any personal context through which to make sense of it.

### The Importance of Follow-up

Follow-up is an integral part of the genetic counseling process, yet there is limited literature focusing on following up non-carrier test results. It may sometimes be erroneously assumed that a non-carrier test result diminishes the need for further input. In this study, some of the participants who received a non-carrier result reported that they would have valued follow-up later in their lives. Although follow-up was not necessarily deemed helpful immediately after testing, the personal significance of a test result has the potential to change over time. This is a challenging issue to address, representing a difficulty with counseling adolescents more generally. Perhaps adolescents may be more inclined to hold back from opening up than adults. This finding supports a previously raised concern that while adolescents may be able to comprehend the meaning of a genetic test, they may not feel confident enough to properly articulate their feelings (Gaff et al. 2006). This may risk underlying anxieties being left unaddressed. Resources need to be considered when assessing the possibility and the benefits of following up non-carrier results.

### Contrasting DMD and BMD

There are some significant differences between the natural histories of DMD and BMD. Symptom progression is more rapid in males with DMD, and life expectancy is significantly reduced. Although it would therefore not be surprising to see the group of DMD participants being more decisive with decisions (actual or theoretical) regarding ending an affected pregnancy, no such difference was identified between the two groups in this study. However, there was only one DMD carrier and only one BMD non-carrier interviewed. This was at least in part due to the availability of carriers/non-carriers through the Manchester Genetic Family Register Service.

### Study Limitations

Bearing in mind that this was a small-scale qualitative study, it is not possible for our findings to be generalized. As the participants ranged in age from 18 to 34, and because some were not yet considering starting a family, it is possible that the significance of some participants’ test results may have not yet become fully apparent to them. Therefore, little can be concluded about the long-term impact of carrier testing for these participants. As the majority of carriers came from BMD families, and the majority of non-carriers came from DMD families, it is difficult to establish any meaningful differences between carriers/non-carriers in this study. None of the participants in this study had gone on to have an affected pregnancy or child at the time of the interviews. It is possible that this group of women self-selected against taking part in the study. This could potentially decrease the perceived significance of being a carrier of either of these two conditions. As women with a prior history of mental health issues were excluded, we cannot draw any conclusions about the impact of adolescent carrier testing in this group of individuals.

### Implications for Genetic Counseling Practice

There are a number of implications for genetic counseling practice that can be extrapolated from this study, though each would benefit from additional research. The research highlights the importance of tailoring the counseling session towards the individual and acknowledging that parents may take an active role in their daughters’ carrier testing process. Our findings also suggest that it could be beneficial to offer adolescents a second appointment prior to having carrier testing. This would allow for more detailed discussion around issues such as disclosing carrier status to future partners. There may also be value in offering follow-up contact soon after testing, and even in contacting both carriers and non-carriers several years after testing.

### Research Recommendations

Further research involving women who have had genetic counseling during adolescence but opted against carrier testing for DMD/BMD would be beneficial. This could potentially elucidate any differences between these two groups of people. Longitudinal follow-up of the women interviewed in this study would provide a valuable insight into the long-term, evolving impact of carrier testing. A follow-up study in 5 or 10 years’ time could help to establish if test results did become more relevant, particularly with regard to reproductive decision-making. Future research could attempt to unpack the differences between the testing process in younger adolescents (i.e., age 13–14) and older adolescents (i.e., age 16–17).

## Conclusion

Genetic testing during adolescence continues to be an area of debate within the genetic community. This study adds to the existing research by exploring the experiences of women who have had carrier testing for DMD/BMD during adolescence, explicitly addressing motivation, the impact of testing, and participants’ experiences of the counseling service provided. This research has demonstrated a minimal negative impact of carrier testing during adolescence, echoing the findings of a number of other studies (Wade et al. 2010; Wehbe et al. 2009; Järvinen et al. 2000). The majority of participants remained positive about having had testing during adolescence, and in the context of receiving support and guidance from their parents, felt that they had made an autonomous decision to undergo testing. The results indicated that carrier status was just one of many factors that influenced future reproductive decision-making.

This study highlights the benefits of tailoring each counseling session to the individual, and the potential importance of follow-up, even in the context of a non-carrier test result. Our findings suggest that adolescent carrier testing for DMD/BMD ought to be accompanied by in-depth pre-test and post-test counseling.

## Electronic Supplementary Material

Appendix 1 (DOCX 14 kb)
