# Supplementary material for: Experiences of Women Who Have Had Carrier Testing for Duchenne Muscular Dystrophy and Becker Muscular Dystrophy During Adolescence
Source: J Genet Couns. 2018 Jul 4;27(6):1349–59. doi: 10.1007/s10897-018-0266-0 (PMC6209047; doi:10.1007/s10897-018-0266-0)
Supplement: Supplementary file 1 — (DOCX 14 kb) [file 10897_2018_266_MOESM1_ESM.docx]

**Appendices**

Appendix 1: Interview Guide

*1. Motivation to request carrier testing during adolescence.*

What was your personal experience of DMD/BMD prior to testing?

Who, if anyone, was affected in the family?

How long had you been aware that you might be a carrier before having the test?

Who initiated discussions about carrier testing?

If asked to look back on that decision, would you do anything differently now?

*2. Impact on a young woman of having a carrier test.*

How did you feel while you were going through the testing process?

How did you feel after you received the results of the test?

Has the result make any difference to what you did (or what you would do) when you have your own children?

*3. Women’s thoughts about the support and counseling they received at the time of testing*.

What do you remember about the genetic counseling process?

Did your discussions with the genetic counselor or the doctor make any difference to your decision to have a genetic test?

Looking back, how satisfied (or dissatisfied) did you feel with counseling process?

Can you say whether genetic counseling helped you to deal with the result?

How did you feel about the amount of information you received during the predictive test process?

Could the genetic counseling process have been improved? If so, how?

Have you had contact with the clinical genetics department, or any other support services, since your result?
